# Damage and protection cost curves for coastal floods within the 600 largest European cities

**DOI:** 10.1038/sdata.2018.34

**Published:** 2018-03-20

**Authors:** Boris F. Prahl, Markus Boettle, Luís Costa, Jürgen P. Kropp, Diego Rybski

**Affiliations:** 1 Potsdam Institute for Climate Impact Research (PIK), Member of the Leibniz Association, Potsdam 14473, Germany; 2 Department of Geo- and Environmental Sciences, University of Potsdam, Potsdam 14469, Germany

**Keywords:** Environmental chemistry, Natural hazards, Climate sciences

## Abstract

The economic assessment of the impacts of storm surges and sea-level rise in coastal cities requires high-level information on the damage and protection costs associated with varying flood heights. We provide a systematically and consistently calculated dataset of macroscale damage and protection cost curves for the 600 largest European coastal cities opening the perspective for a wide range of applications. Offering the first comprehensive dataset to include the costs of dike protection, we provide the underpinning information to run comparative assessments of costs and benefits of coastal adaptation. Aggregate cost curves for coastal flooding at the city-level are commonly regarded as by-products of impact assessments and are generally not published as a standalone dataset. Hence, our work also aims at initiating a more critical discussion on the availability and derivation of cost curves.

## Background & Summary

Establishing the relation between economic loss and flood height is a crucial step in the elaboration of flood-damage assessments in coastal zones, be it at global^1^ or city scales^2^. Such relations take the form of damage cost curves and are used by researchers as an essential ingredient for the estimation of economic loss due to sea-level rise and the associated intensification of storm surges^3^. In a wider sense, they constitute the starting point for the appraisal of the benefits of adaptation in light of climate change^4^. Despite their pivotal role in the workflow leading to economic assessments of coastal impacts and adaptation, academic literature has put little scrutiny on the derivation of cost curves or their comparison.

Mainly seen as a by-product of the impact assessment itself, damage cost curves are generally not published as a standalone dataset. At the case-study scale, damage cost curves are rarely derived in a consistent manner, with regard to the data sources for assets at risk and the employed cost methodology. These factors severely limit the comparability of cost results and renders the synthesis of knowledge embedded in such curves a daunting task. In order to advance the comparability of forthcoming impact assessments of coastal flooding, and to initialize a more critical discussion on the availability and development of cost curves, this work releases a consistently derived set of macroscale damage and protection cost curves for the 600 largest European coastal cities. We employ the term macroscale to indicate that the curves are obtained for the entity of the city, and also to differentiate our cost curves from depth-damage functions for single elements at risk (also termed microscale)^5^.

Given the local character of damages and adaptation^6^, the data and processes chosen for the elaboration of the cost curves must, on the one hand, contain a certain degree of spatial detail to capture the city/landscape specificities that play an important role in shaping the magnitude of the impacts. On the other hand, it is mandatory for the objectives of this work - comparability and transferability - that data retains a certain level of universality in order to be generically available. The balancing of these two opposing premises shaped to a large extent the primary data choices made in this study.

Our approach differs from previous studies estimating potential damage from coastal flooding by employing land-use information as the basis to approximate the location of assets, rather than using population coupled with GDP per capita, as found in similar studies^7,8^. The use of land-use information guarantees that our main object of analysis is the built-up city itself and not a less consistent approximation based on regional political or socio-economic consideration. Regarding hydrological modeling, we make use of a high resolution digital elevation model for Europe for determining areas at risk from flooding. To generate inundation maps, we employ a simple though commonly used static-inundation model that only accounts for hydraulic connectivity. Because we are interested in determining the rise of direct monetary damage with increasing flood height, we raise the flood height in small incremental steps up to a maximum of 12 m. This range was chosen to cover potential extreme sea levels both under current and future conditions^9^.

The benefits of the damage cost curves to the scientific community are manifold. At the foremost, the comparability between alternative cost curves for different cities will be improved. The large number and size range of cities investigated makes room for further quantitative insights on the characteristics of cost curves. Because the provided cost curves embed in themselves a measure of asset vulnerability they only require information of typical or expected flood events for a given city in order for damage assessments to be performed. As regional and global datasets on sea-level rise and storm surges become available at increasing resolutions^10–12^, the existence of a consistent database of damage cost curves at city-scale will unlock new potential for the systematic analysis of coastal damage from sea-level rise in cities.

Unique to this work is the evaluation of the protection needs of urban land within a city at increasing levels of coastal flooding. The protection cost curves describe the relation between the construction cost of coastal protection (whose costs are approximated by those of building dikes) and the designated protection height. The curves provided allow to contextualize the adaptation efforts expected in each city, while at the same time providing impact researchers with the underpinning information to run comparative assessments of costs and benefits of coastal adaptation.

## Methods

In this study, we derive macroscale damage and protection cost curves for European coastal cities. The cost curves are a product of combining information about the orography and land cover of a city and merging these with statistical information on land use and its associated monetary value.

It is relevant to underline that only European cities are considered, given the availability of some datasets and the restrictions imposed, namely a consistent classification of land cover and land use. Table 1 summarizes the sources and characteristics of the data used. In the following, we disclose each step involved in the estimation of the macroscale damage and protection cost curves and also introduce the relevant datasets in detail.

### Step I: Inundation modeling

With a horizontal resolution below 100 m, recent satellite-based digital elevation models (DEM) have received much attention in flood modeling and have been established as an important data source for large-scale damage assessments^8,13–15^. Following this approach, we make use of the EU-DEM^16^ to model hypothetical inundation at the European coast at a horizontal resolution of 25 m (see Table 1).

**Figure 1:**
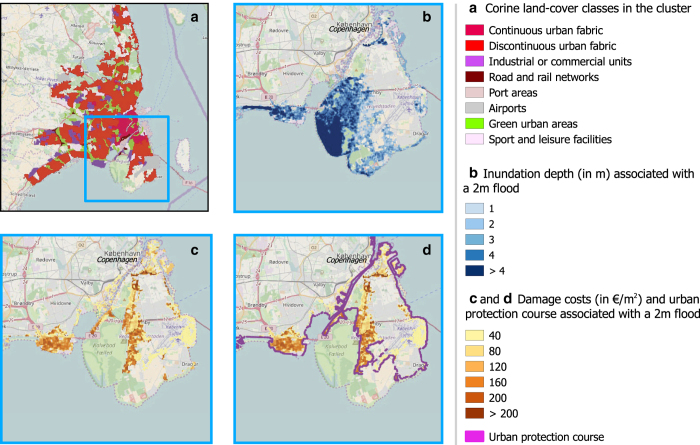
Inundation, flood damages and protection needs for the urban cluster of Copenhagen at 2 m flood height. (**a**) Identification of the urban cluster and its CORINE land-cover composition. (**b**) Inundation depths as calculated by the flood-fill algorithm. (**c**) Spatial distribution of damage costs within the urban cluster. (**d**) Urban protection course (UPC) of flood defenses (shown in purple) required to avoid the estimated damages.

Employing a static inundation scheme^17^, we estimate areas that are hydraulically connected with the sea at a given sea level. A flood-fill algorithm using 8 nearest neighbors^18^ determines grid cells that are flooded at a presumed flood height. For each of these, we store the corresponding inundation depths. The whole procedure is repeated for flood heights between 0 m and 12 m in steps of 0.5 m. An example for the inundated area and the corresponding inundation depths is given in Figure 1b for a 2 m flood at the city of Copenhagen. It is acknowledged that this approach disregards the effect of natural or artificial flood barriers that may not be resolved by the DEM. Furthermore, the static inundation scheme provides an upper estimate of the flooded area. Further discussion of this approach is given in the Technical Validation section.

### Step II: Identification of cities from land cover

In order to ensure a consistent identification of coastal cities, we use a systematic procedure to identify the spatial extent of continuous urban land located at the coasts of Europe. For this purpose, we apply a cluster algorithm^19,20^ to the CORINE 2012 Land-Cover dataset^21^ (CLC, see Table 1). We thereby follow the definitions of Urban Morphological Zones^22^ (UMZ), which define the maximum distance between connected cells and the specific land-cover classes included (listed as columns in Table 2).

Due to the coarser resolution of the CLC data compared to the EU-DEM, some cluster grid cells may protrude into the open sea. In order to avoid such artifacts, we trim the CLC-based cluster at the coast by considering additional vector data for the European coastline^23^. We retain only those parts of a CLC grid cell that lie behind the coastline or that overlap with a DEM cell that shows a positive elevation above MSL.

**Figure 2:**
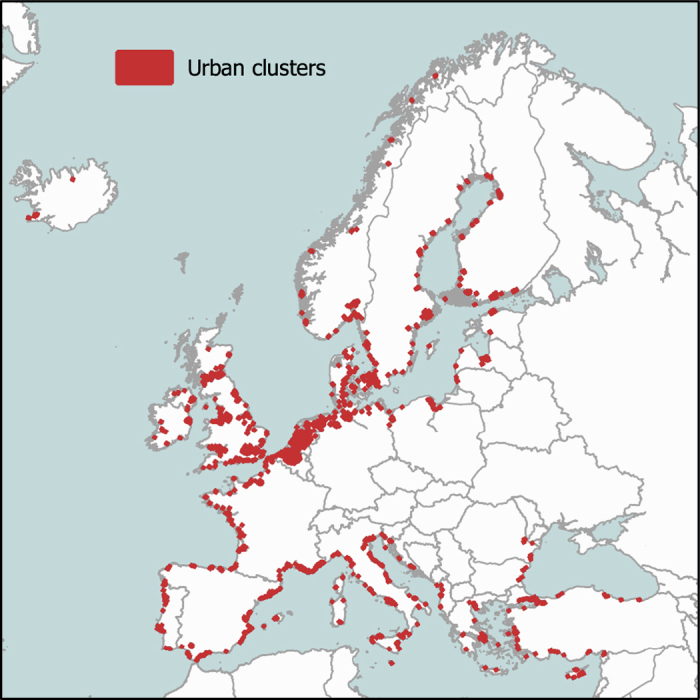
City clusters considered in this study. We provide damage and protection cost curves for the 600 largest urban clusters spread along the European coast. For better visibility on the map, the cluster bounds have been enlarged.

We keep only those city clusters that are either directly adjacent to marine waters or located with at least 10% of their area in low-lying coastal zones (defined as no more than 10 m above MSL). From these coastal clusters, the 600 largest (by area) are selected for further investigation. A breakup of the number of selected clusters per country is given in Table 3 and their locations at the European coast in Figure 2.

It should be noted, that the clusters do not correspond to administrative boundaries but represent connected areas of urban land cover. Hence, it is possible that the algorithm combines adjacent cities within one urban cluster. For instance, our analysis provides a joint cluster for the so-called Flemish Diamond, an urban agglomeration comprising the major cities of Antwerp, Ghent, Louvain, and Brussels. In two cases (i.e. the Flemish Diamond and Manchester/Liverpool) we provide further sub-clusters that relate to separate cities, each of which individually qualifies as coastal cluster according to the above criteria.

For the following steps, the cluster boundaries are used as a mask to extract the corresponding land cover and inundation information. We give an example of the results for the city of Copenhagen in Figure 1a.

### Step III: Inference of land use from land cover

So far, the clusters boundaries define the location of assets at risk. However, the estimation of damage cost requires information about the quantity and the economic value of these assets (exposure). We estimate exposure on the basis of the economic value of land use. For this purposes a statistical mapping between land-cover and land-use classes is required. The mapping is created from raw data of the 2015 Land Use/Cover Area Frame Statistical Survey^24^ (LUCAS). Specifically, we statistically relate observed land use and CORINE land-cover classes over all geo-referenced observation within the LUCAS data. Finally, every land-cover class is allocated the average fraction of the land-use classes. Table 2 shows the derived mapping between the considered land-use classes and the land-cover classes that are included in the urban clusters.

For this study, we limit the considered land uses to the classes residential, commercial, industrial, road transport, and agriculture, as well as unused land (cf. Table 2) and disregard other LUCAS land-use classes. This choice is motivated by the availability of microscale depth-damage functions, which are not available for other land-use classes. As a consequence, we can only account for an overall share of 0.82 of the aggregated cluster area. Details on the *considered share*, i. e. the fraction of a cluster cell that can be attributed to the considered land uses, are given in Tables 2 and 3.

### Step IV: Application of microscale depth-damage functions

**Figure 3:**
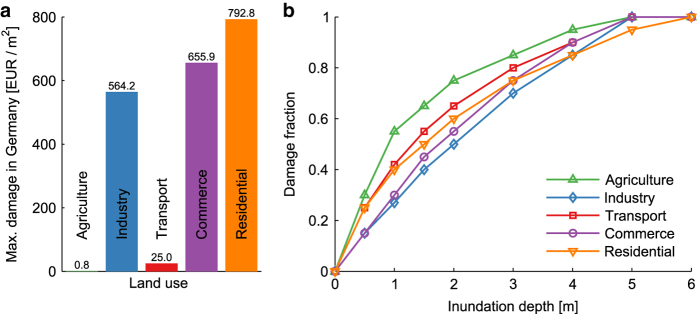
Depth-damage functions for different land uses. (**a**) Average maximum damage per m^2^ for Germany (inflation adjusted). (**b**) Relative depth-damage functions for each land use. Both maximum damage and relative depth-damage functions have been adapted from Huizinga^25^.

Microscale depth-damage functions are used to convert the grid-based information on inundation depth and land use to monetary damage. The depth-damage functions are adapted from publications by Huizinga *et al.*^25,26^, who provide country-specific data for five different land-use classes. For each country, the data contain land-use specific values for the average maximum damage per m^2^. These values are used to calibrate relative damage functions that were derived at the European level for the considered land use^25^. For the example of Germany, we show the inflation-adjusted average maximum damage in Figure 3a. The generic relative depth-damage functions are shown in Figure 3b.

Huizinga^25^ relates the average maximum damage per land use to the economic output of a country measured in terms of GDP per capita using Purchasing Power Standards (PPS) for the year 2004. In the lack of a more recent land-use valuation, we adjust monetary estimates to 2016 price levels by using historic inflation rates based on the consumer price index^26^. For most countries the inflation rates were obtained from Eurostat^27^, for Albania and Montenegro from the Worldbank^28^.

Switching from land use to land cover data, we infer the microscale damage function *d*_*i*_ for a land-cover class *i* as a weighted sum of the corresponding damage functions *d*_*j*_ for land-use classes *j*,

(1)di(h)=∑jwi∧jdj(h),

where *h* denotes the inundation depth and the weight *w*_*i*∧*j*_ represents the observed frequency of finding both *i* and *j* attributed to the same grid cell. The weight is taken from the statistical mapping between land cover and land use shown in Table 2.

Knowing the land-cover class and the inundation depth for each grid cell, we can use Equation (1) to calculate monetary damage for each grid cell within a city cluster. As an example, damage costs per m^2^ for a hypothetical 2 m flood in Copenhagen are shown in Figure 1c.

### Step V: Macroscale aggregation of monetary damage

In this step, we combine the information on the urban cluster with the modeled inundation data and the microscale damage functions. For each urban cluster the monetary damage at a specific flood height is given by the sum over the damages for each of the inundated grid cells^29^. Precisely,

(2)D(x)=∑ndi(n)(hx,n),

where *D* is the total damage in the considered cluster at flood height *x*. The index *n* runs over all grid cells in the cluster and *d*_*i*(*n*)_ is the microscale damage function for the corresponding land-cover class in cell *n*. The inundation depth *h*_*x*,*n*_ is obtained from the inundation model in Step I and is dependent on the overall flood height *x* and the location of grid cell *n*. Following this approach, we obtain the flood damages for all 600 urban clusters at all flood heights x=0m,0. 5m,1m,…,12m.

### Step VI: Estimation of protection costs

Using the results of Steps I and IV we determine the protection needs for a given urban cluster in terms of an *urban protection course* (UPC). The novel terminology has been chosen deliberately to stress the hypothetical nature of this product and its dependence on the urban cluster. The UPC can be understood as the entirety of all flood defense measures, whether they are dikes, sea walls or any other artificial construction.

In order to obtain the UPC, we need to identify grid cells, where the construction of a flood defense structure is theoretically required to avert the potential damage at a given flood height. First, we extract the outline of the city cluster by finding those cluster cells that belong to the cluster and are adjacent (in the sense of the 8 nearest neighbors) to the surrounding land, whereby the surrounding land excludes grid cells that are entirely enclosed by the city cluster (i.e. holes). Second, cells on the outline are identified as part of the UPC if they suffered a potential flood damage during Step IV.

The required height of the protective structure at an identified UPC cell is defined as the difference between the cell's elevation and the considered flood height. The required length of the protective structure is given by the edge length of the cell itself. An example for an UPC in the city of Copenhagen at 2 m protection height is given in Figure 1d.

It is important to distinguish between the UPC, which is a purely hypothetical protection course around an urban cluster, and existing or planned flood protection which typically protect wider areas. Crucially, the UPC shall not be misunderstood as a basis for engineering decisions on coastal defense measures due to its known limitations, e. g. of the underlying DEM and the simplicity of the hydrostatic approach. A critical discussion of the UPC is included in the Technical Validation section, where we also compare the UPC and existing protection measures for the city of Hamburg.

Since the UPC represents a compilation of potentially several different flood defense measures, its construction costs can only be estimated approximately. Since empirical costs estimates for coastal defense measures are only sporadically available on case study level, construction costs are commonly assessed via a unit-cost approach^30–32^. We employ unit costs for dike construction (cost of constructing or raising a dike per unit length and height)^31^ as a general cost proxy for the UPC. Protection costs are obtained by applying the unit cost to the product of protection height and length of each grid cell and summing over all grid cells belonging to the UPC. Repeating this process for every protection height ω=0m,0. 5m,1m,…,12m, we obtain individual cost curves for all cities considered. We provide both a low and a high cost scenario^31^ that reflect the high uncertainty^32^ of empirical dike construction costs.

Analogous to the preparation of the damage costs, we stratified the protection costs for the different European countries by calculating the difference in Purchasing Power Standards^25^. Subsequently, regional protection costs were inflation-adjusted to 2016 price levels^26^ analogous to the damage estimates in Step IV.

### Code availability

All data products were computed using Matlab R2014a. For computational efficiency, the Matlab functions for generating the city clusters and the inundation maps were coded in C and compiled with the Microsoft Windows SDK 7.1 C Compiler. Raster data were prepared either directly in Matlab or by using the Geospatial Data Abstraction Library 2.2.1 (GDAL). Any maps were generated with QGIS, version 2.18.

In the Supplementary Information we provide a stand-alone example of the Matlab codes and data to generate the damage and protection cost curves for the Copenhagen urban cluster. Further codes are specific to our computational architecture and can be made available upon request.

## Data Records

All data products that were derived and described in this work are publicly available at the PANGAEA repository [Data Citation 1] under the Creative Commons Attribution 3.0 Unported license. In detail, [Data Citation 1] provides four distinct datasets:

- [Data Citation 2] provides binary masks for each coastal city cluster derived from CORINE land-cover and EU-DEM data. The dataset comprises individual raster files (GeoTIFF) for the 600 largest clusters, as well as additional files for the 8 sub-clusters of the Flemish Diamond and Manchester/Liverpool megaclusters. The archive further contains auxiliary metadata, namely: The name and geographical coordinates of the clusters, as well as the area of the clusters in km^2^.
- [Data Citation 3] provides the direct monetary damage (million € in 2016) for each of the considered city clusters at flood heights varying from 0 to 12 m in 0. 5 m steps. Although existing flood protection is not considered, we propose an approach to take into account the impact of flood protection in the Usage Notes section.
- [Data Citation 4] provides lower bound cost estimates (million € in 2016) for potential protection at each of the considered city clusters. Costs are absolute figures for the construction of the UPC at design flood heights varying from 0 to 12 m in 0. 5 m steps. Note that existing flood protection is not considered in the cost estimates, but may still be incorporated based on simple assumptions (cf. Usage Notes).
- Analogous to [Data Citation 4], [Data Citation 5] provides upper bound cost estimates (million € in 2016) for potential protection at each of the considered city clusters.

In the following, we provide a detailed account of each of the individual datasets.

### Cluster masks

In [Data Citation 2] derived cluster masks are provided for each of the 600 largest urban clusters located at the European coast. The spatial distribution of the clusters is shown in Figure 2. An example of a cluster mask applied to CORINE land-cover data can be found in Figure 1a for the city of Copenhagen. The 600 clusters are located in 28 European countries, with the largest exposure in the Netherlands, Germany, UK, Italy, and France. A detailed breakup of the number of clusters per country and affected monetary value is given in Table 3.

Within the 600 clusters there are two which comprise two or more major cities. These are the Flemish Diamond of Brussels, Antwerp, Ghent, and Louvain, as well as the cluster of Manchester/Liverpool. These were disaggregated into 8 subclusters that are provided alongside the original clusters. All further results are available for both the original clusters and the disaggregated coastal sub-clusters.

While the employed damage functions account for the predominant land use in urban areas, not all land use could be accounted for. We find that our method can attribute an average of 82% of the cluster area to accountable land use. Other land uses are not considered due to the unavailability of value estimates and damage functions. Tables 2 and 3 provide a breakup of the considered share of the cluster area per land-cover class and per country, respectively. Concerning land cover, we find that all land-cover classes associated with built-up areas can be attributed by at least 80% and only green urban areas and sport and leisure facilities are covered to a lesser extent. Across countries the considered share remains approximately constant, with small fluctuations between 0.77 and 0.86.

When considering the affected values (exposure) at a hypothetical 5 m flood height, the total exposure accumulated within the coastal clusters equates to more than half of the total urban exposure within Europe. In numbers, we find that 58% of the total exposure are due to the top 600 clusters.

### Damage cost curves

**Figure 4:**
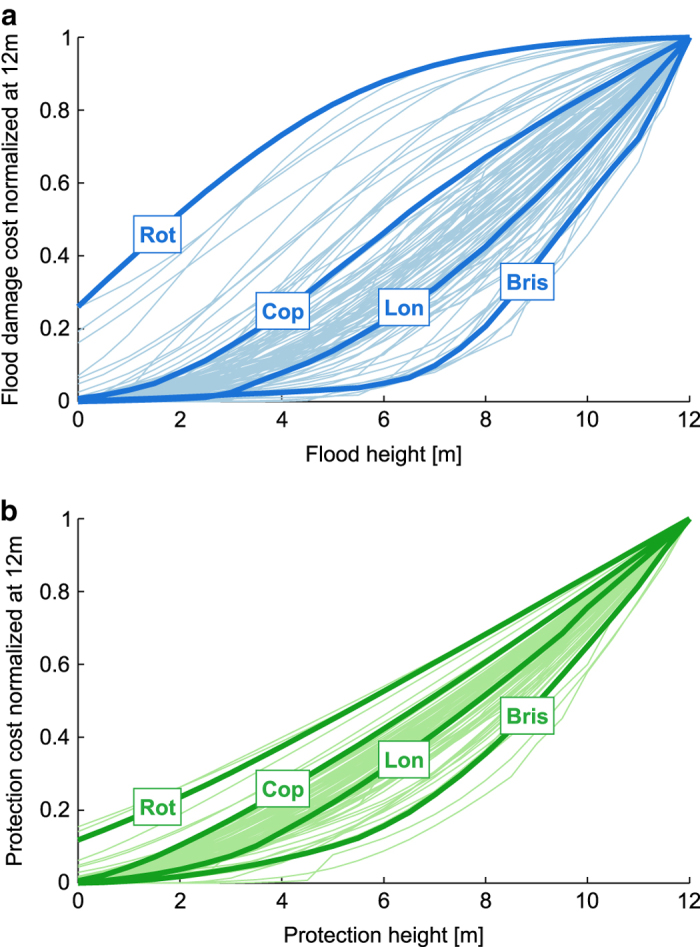
Damage and protection cost curves for top 100 urban clusters with the largest area. (**a**) Normalized damage curves, where each curve has been divided by the damage at a hypothetical 12 m flood height. (**b**) Normalized protection cost curves, where each curve has been divided by the protection cost at the 12 m height. The curves for the cities of Rotterdam (Rot), Copenhagen (Cop), London (Lon), and Bristol (Bris) have been highlighted.

[Data Citation 3] provides estimated cost curves for direct monetary damage for each of the 600 clusters as well as the 8 subclusters. The curves for the largest 100 clusters are shown in Figure 4a. Given the strong differences in the absolute value of estimated damages, the curves are normalized by the maximum damage at 12 m flood height. Probably most eye-catching is, that some curves exhibit a considerable damage at a 0 m flood height, which at first appears counter-intuitive. This effect is explained by the fact, that our approach considers the potential damage costs in the absence of any coastal protection measure. Accordingly, low-lying cities such as Rotterdam in the Netherlands can already be affected at 0 m flood height. Apart from that, it can be seen that almost all curves exhibit a convex increase for low and moderate flood heights. Furthermore, the damage cost curves exhibit inflection points for various city clusters and even approach saturation in a few cases. This behavior arises when most parts of the city have already been inundated and there is a diminishing increase of the flooded urban area. The saturation is seen most prominently in the example of Rotterdam.

Overall, the majority of curves exhibit a moderately convex (e.g. London), or slightly sigmoid (e.g. Copenhagen) behavior over the entire range from 0 to 12 m. As for the example of Bristol, cities that are mostly located on elevated grounds exhibit a strongly convex damage cost curve with no saturation for the flood heights considered.

### Protection cost curves (low/high)

For [Data Citation 4] and [Data Citation 5] approximate protection cost curves were estimated for each of the 600 clusters as well as the 8 subclusters. We obtained a lower and an upper bound of protection costs for each city, which result from applying the lower and upper unit cost figures, respectively (cf. Step VI in the Methods section). Analogous to the damage cost curves, we show the protection cost curves (upper bound) for the 100 largest clusters in Figure 4b. Similarly to the damage costs, some protection cost curves exhibit a positive offset at zero protection height. Again, this results from the fact that existing protection is not considered and therefore low-lying cities may experience hypothetical flooding below mean sea level. In contrast to the damage costs curves, the protection costs show a convex increase throughout. Furthermore, they do not saturate but converge to a linear function of protection height. The linear increase arises, when the protection course spans the entire city and costs arise merely due to a gradual heightening of dikes. This is clearly seen for the low-lying example of Rotterdam, whereas the elevated city of Bristol exhibits a super-linear increase throughout. Since both damage and protection cost curves are dependent on the extent of the flooded area, we see the same ordering for the four example cases.

## Technical Validation

The feasibility of a technical validation is limited given that the damage and protection cost curves can only provide a characterization of potential cost under the assumption of no existing protection. Even if existing protection levels were modeled, empirical cost estimates at the city level remain scarce and may not be consistent in terms of the considered city areas and asset values. (A general approach to incorporate existing protection levels for risk assessment is described in the Usage Notes section.)

In order to address these limits, we focus our validation on three different aspects. First, we undertake a critical discussion of the caveats implicit in the data and the model choices of this work. Second, we identify comparable damage cost curves for 17 coastal cities in the scientific literature and compare these against our estimates. Third, we discuss the characteristics of the UPC against existing protection on the example of Hamburg.

### Critical discussion of the modelling approach

#### City boundaries

The definition of city boundaries is a long-standing problem in any field studying cities^33^. As population data is collected on the administrative level, the apparent choice is to simply rely on those definitions. Administrative boundaries, however, in general disagree with the common sense spatial extent of cities, so that definitions of metro-regions aim for consistent density and commuting thresholds^34^.

Since damages primarily arise from urban infrastructure, we employ a physical city definition which closely matches asset exposure and potential damage proxies. According to our definition, cities are spatial clusters of urban land cover, i. e. any two neighboring urban grid-cells belong to the same city cluster.

By following the EEA's definition of urban morphological zones^22^, we employ a systematic and reproducible procedure to extract city boundaries from land-cover data. Since land-cover data is also the source of our damage proxy, our approach assures consistency between the city boundaries and the estimated monetary damage.

#### Assets at risk

Given the large number of city clusters considered and the continental scope of analysis, approximating the economic value of assets at risk is bounded by a number of caveats. Unlike local studies in which asset value is available at the building level^18^, continental and global assessments need to approximate asset values using economic information with a comparably coarser resolution. Economic proxies for asset exposure (e.g. GDP per capita) are typically downscaled using more detailed geographical information. For this purpose, recent studies have employed remote sensing data for population^4,7,8,35^, land use^35^, land cover^36^, or night light^37^ information.

Fundamentally, our study is based on the average maximum damage for each land use per country^25^. Where country averages are not available, European values are scaled to the country level by the ratio of national over European GDP per capita in Purchasing Power Standards, thus reflecting the differences in economic output^25^. This procedure results in monetary estimates that are homogeneous for each country.

As with similar studies^7,8,35^, these national estimates do not differentiate between relatively poor or wealthy regions or between more rural or strongly urbanized settlements. While some population-based studies have employed a factor of 2.8 to approximate asset values from national GDP per capita^4,8^, others have used a factor of 5 to account for increased affluence in cities^7,35^. The higher factor for cities can be motivated from the fact that GDP scales super-linearly with the size of the population^38^. If sub-national estimates of GDP per capita are available for a given city, the damage cost curves provided here could simply be rescaled by the ratio of city GDP over national GDP.

In our approach the maximum damage of a grid cell is dependent on land use and local GDP per capita but independent of population density. This is a major difference to population-based approaches where total assets are assumed to be proportional to the local population. This aspect has two main consequences. On the one hand, our approach could overestimate potential damage in low-density areas. Due to the high 100 m resolution of the underlying CORINE data, we believe this to be a minor effect. On the other hand, studies based on population and GDP per capita may fail to accommodate for high-rise buildings in city centers, which are typically less affected by flood damage in relation to their total economic value. They may also systematically undervalue industrial and commercial sites which are not resolved explicitly. In contrast, our approach identifies and evaluates different types of built-up areas by land use and, thus, yields a more accurate spatial distribution of damages. Moreover, the use of land use can in principle be more appealing to spatial planners and adaptation practitioners.

It should be noted that the allocation of average fractions of land use to the CORINE land-cover data could be a source of error for individual damage cost curves. Locally, the composition of land use per CORINE cell could be different from the statistical average. Generally, this random error is expected to become less significant at higher flood heights where more and more cells are affected, and it should be negligible for a large-scale assessment on the national or European scale.

#### Microscale depth-damage functions

Despite the existence of a considerable amount of functions relating flood depth and relative damage at the building-level their overall shape is remarkably similar—a sub-linear rise of damage for the initial flood depths followed by a slow deceleration of damage and saturation for high flood depths. The microscale depth-damage functions used in this work (Figure 3b) apply to the average damage per m^2^ for different land uses, but they comply with the general shape of damage functions at the building level. While the maximum damage value per land use is broken down for each country according to its GPD in terms of Purchasing Power Parity^25^, the shape of the depth-damage functions remains homogeneous across Europe. This implies that damage effects stemming from country and behavioral-specific features are not captured. For example, differences in construction materials^39^ and the existence of an underground floor^40^ significantly influence the shape of the relative damage function. Furthermore, the existence of flood-adapted use and adapted interior fitting are reported to have a significant effect in shaping damages^41^ and hence the modification of the relative damage function.

#### Inundation maps

Inundation maps produced are a explicit spatial representations of hypothetical flood events and thus define both the extent of the flooded area as well as the water height (inundation depth) at each individual point. Conventional approaches range from simple static inundation schemes to computationally demanding dynamic inundation modeling^9^.

Static inundation schemes, sometimes also called bathtub models, are often used for large-scale flood damage assessment, where computational time becomes a limited resource^4,8^. The comparison of different inundation schemes shows that static models may overestimate the flooded area^9^. The overestimation is due to the fact that static inundation schemes assume infinite water volume for immediate discharge during the flood event, disregarding limitations due to terrain roughness, narrow or constricted openings, and flow velocity in general.

The choice of the DEM and its resolution has a substantial effect on the accuracy of inundation models. In this context, many studies have demonstrated the benefit of using airborne light detection and ranging (LiDAR) data, which offers high horizontal resolution and vertical accuracy, as well as the capacity to separate bare-earth from built structures and vegetation^13,14,42^. However, since LiDAR data is not consistently and often not freely available, large-scale flood damage assessment relies on satellite based observations.

Recent large-scale studies have employed the shuttle radar topography mission (SRTM) DEM^4,8^, whose general utility as a source of global terrain data has been noted^42^. Comparative studies show significant differences between SRTM and LiDAR for inundation mapping^14^, but also indicate that the differences may be within the accuracy that is typically associated with large-scale flood studies^13^.

This study employs the EU-DEM^23^, which is largely SRTM based and would thus be expected to provide similar accuracy. A first study comparing SRTM and EU-DEM products for flood modeling did not find significant differences^15^. However, since all elevation data were averaged to the same resolution as SRTM, the benefit of the higher resolution of the EU-DEM was not assessed.

#### Costs of protection

Empirical cost estimates are scarce and generally not available for the entirety of flood defense measures around a specific city. Hence, a direct validation of protection cost against reported figures is not feasible and, as a consequence, we discuss the costing and the location of flood defenses separately.

In our work we employ empirical estimates of protection cost that reflect the costs per 1 m increase in dike height^31^. Derived from actual project data, the unit cost include material and construction costs, as well as the costs for design, taxes, and fees^31^. Strictly, the unit-cost approach only applies to the incremental cost of raising dikes, such that absolute cost figures are potentially unsubstantiated. However, the unit-cost approach is supported by the finding that empirical costs for Canadian and Dutch dikes can be approximated by linear cost functions^32^.

Costs for protection measures other than dykes are not considered in this work. Estuaries and in particular harbor cities require adaptable storm surge barriers, such as flood gates, in order to allow passage and water exchange at ordinary water levels. The construction costs of storm surge barriers are significantly higher than those for sea dykes. Based on empirical estimates for the construction costs of flood barriers^31^ we compute a range of €67.1 million to €259.0 million per m height and km width. These values are a factor 5 to 12 greater than the unit costs for sea dykes in urban areas, which have been estimated at €15.5 million and €22.4 million in ref. 31. However, the feasibility and cost of constructing flood gates critically depend on the local conditions and require the consideration of in-situ hydrological, bathymetrical, ecological, and economical information—information that is not systematically available at the European level. Additionally, urban drainage systems (e.g. pump stations) play an important role for the removal of stormwater. Again, such facilities are highly dependent on in-situ characteristics and could not be considered here.

Under the unit-cost assumption, the shape of the estimated protection cost curves is determined solely by the length and height of the required flood protection. This information is provided by the UPC, which is based on the the city cluster that defines the urban land cover attributable to the city. As such, the UPC can be compared to the existing protection measures deployed within the city. However, the actual protection measures and the UPC differ in their objective. For consistency with our damage estimation, the UPC was designed to protect those urban areas which would otherwise suffer damage. As such, the UPC does not reflect economic considerations or protection needs beyond city cluster boundaries. In contrast, the processes leading to actual protection are very complex and might involve a local political discourse and constitute a compromise between economic and social aspects. The implicit decision process likely considers the city and its surroundings such that the protection in place potentially deviates from the UPC.

Given the high variance in empirical protection costs^32^ and the specific assumptions of the UPC, it becomes evident that the monetary estimates of the protection costs should be considered as indicative. However, courtesy to the consistent approach, the estimates are well suited for differential analysis of protection costs across different cities.

### Comparison of our results with damage cost curves in the literature

While being of great value for risk assessment, macroscale damage curves are typically being considered as an intermediate result of the damage assessment and often not published. This fact unnecessarily hampers the comparability and accessibility of flood risk assessments and hinders the validation of such curves.

We consider only one study^8^ comparable to the one presented, both in regard to the number of cities considered and methodological homogeneity. This other study undertakes a risk assessment for the 136 major coastal cities worldwide. There is an overlap with our study for 17 urban agglomeration within Europe. The supplementary material to ref. 8 contains raw data and computational codes from which the implicit damage cost curves can be constructed. Employing no additional data, we hence reconstructed the damage cost curves in the original 2005 USD. For comparability, these values were converted to EUR and adjusted to 2016 values.

If available, one would prioritize the comparison of the damage cost curves in this work with those derived from case-study investigations. We do so for the city of Copenhagen, where a monetary damage curve has been disclosed^2^.

**Figure 5:**
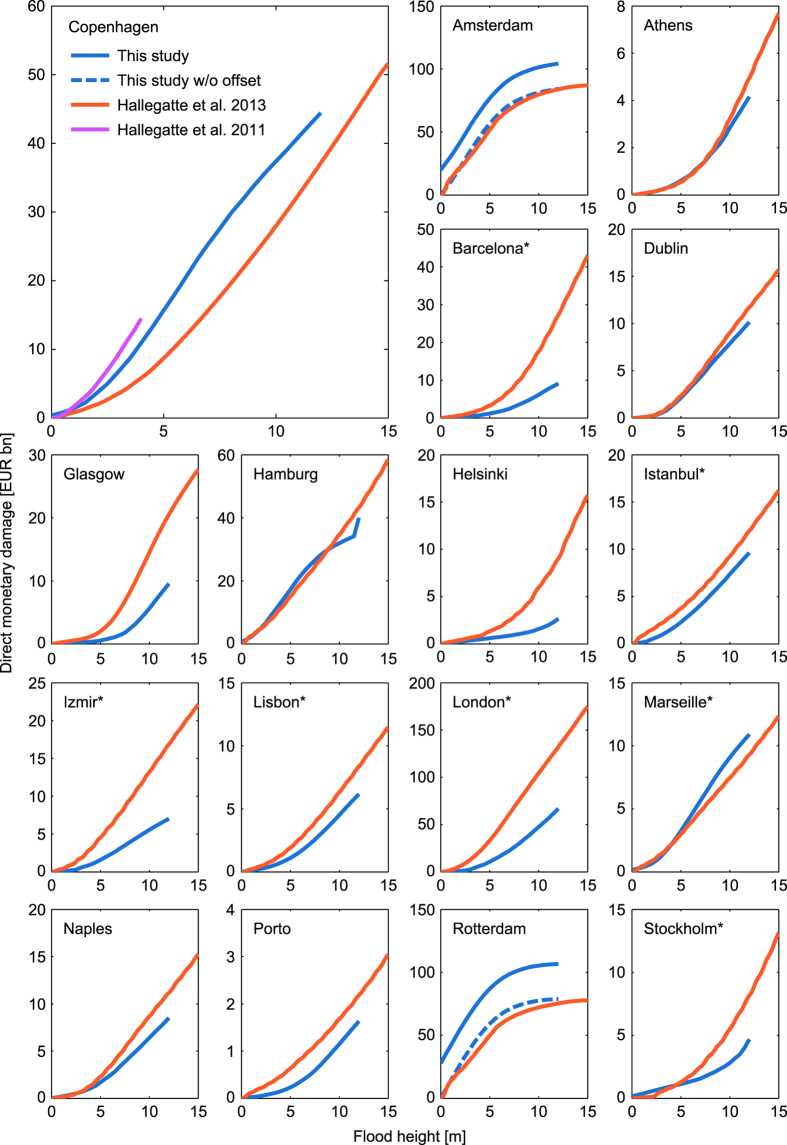
Comparison of the estimated damage curves with other studies. Loss estimates for 17 European cities were obtained from Hallegatte *et al.* 2013 (ref. 8). An additional damage curve for Copenhagen was obtained from Hallegatte *et al.* 2011 (ref. 2). An asterisk (*) identifies those cities, where more than one coastal city cluster was combined to match the area considered in ref. 8.

Figure 5 provides a comparison between our damage cost curves and the results obtained from literature^2,8^. Despite the use of distinct datasets and methodology for asset estimates in the included studies (cf. Table 4), we find a remarkable agreement between cost curves for the cities of Athens, Dublin, Hamburg, Marseille, and Naples. Furthermore, a very good match for Amsterdam and Rotterdam after subtraction of the damage offset at 0 m flood height. This shift is explained by the fact that in ref. 8 inundation is considered only above 0 m elevation, neglecting the possibility of low-lying areas beneath the mean sea level. For the remaining cities we note strong deviations at low flood heights below 3 m and deviations up to a factor of 4 at higher flood heights.

The large spread of the different damage cost curves for Copenhagen gives an indication of the typical variability among damage cost curves. Table 4 shows that the external studies^2,8^ differ only with respect to the asset exposure and the choice of vulnerability/depth-damage functions. For example, the exposure at a 5 m flood height equates to €4.28 billion in ref. 2 and €16.14 billion in ref. 7, both in 2005 EUR (compared to an estimated exposure of €29.70 billion at the 5 m level in this study). However, despite the considerably larger asset exposure, the curve from ref. 8 shows not ≈ 4 times but a mere ≈ 1/3 of the losses of that of ref. 2. At the lack of more detailed information, this effect must be attributed to a strong difference between the proprietary vulnerability functions and the depth-damage functions. The fact that both types of microscale damage functions are typically derived from empirical data urgently shows that absolute estimates from damage assessments may be significantly biased.

**Figure 6:**
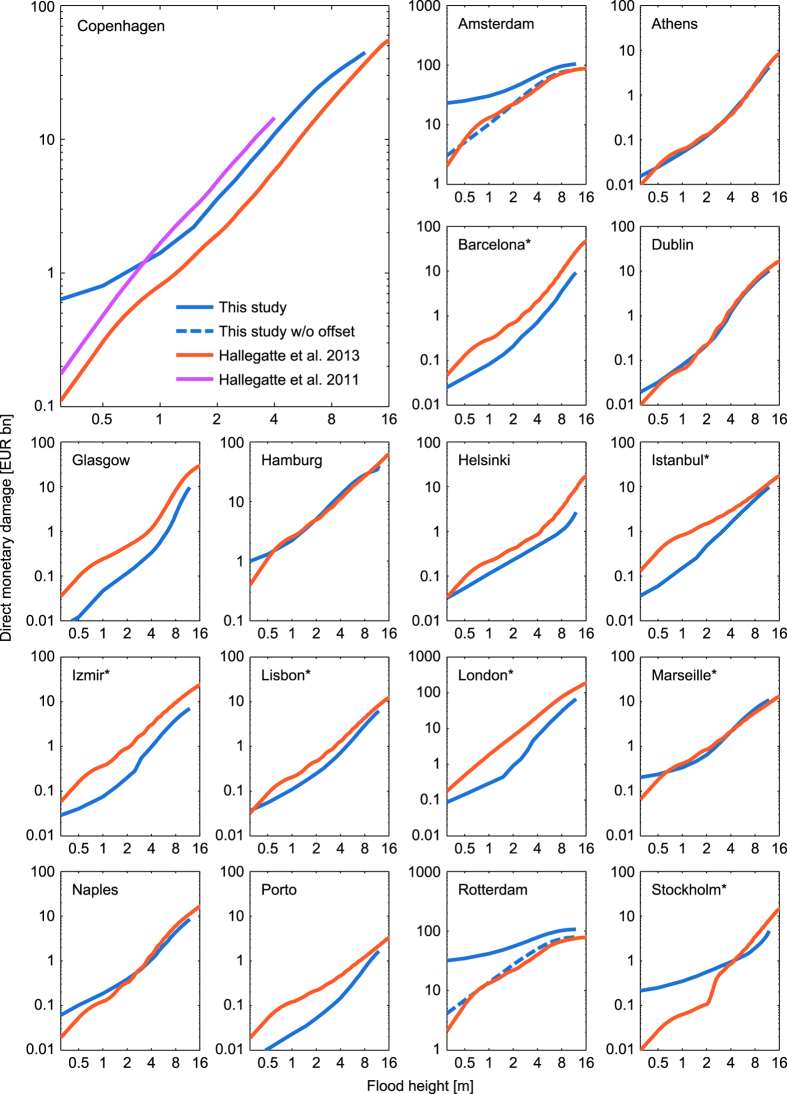
Log-log comparison of the estimated damage curves with other studies. This figure is analogous to Figure 5 but in log-log scale in order to better resolve the lower ranges. An asterisk (*) identifies those cities, where more than one coastal city cluster was combined to match the area considered in ref. 8.

Nonetheless, Figure 6 suggests that the different damage curves are nearly parallel on a log-log graph in the majority of cases. If parallel, curves differ only by a constant coefficient, i. e. are subject to a simple multiplicative bias. This implies that relative differences (e. g. percentage increases) are more similar and robust with regard to model choice.

### Comparison of the urban protection course with existing protection on the example of Hamburg

**Figure 7:**
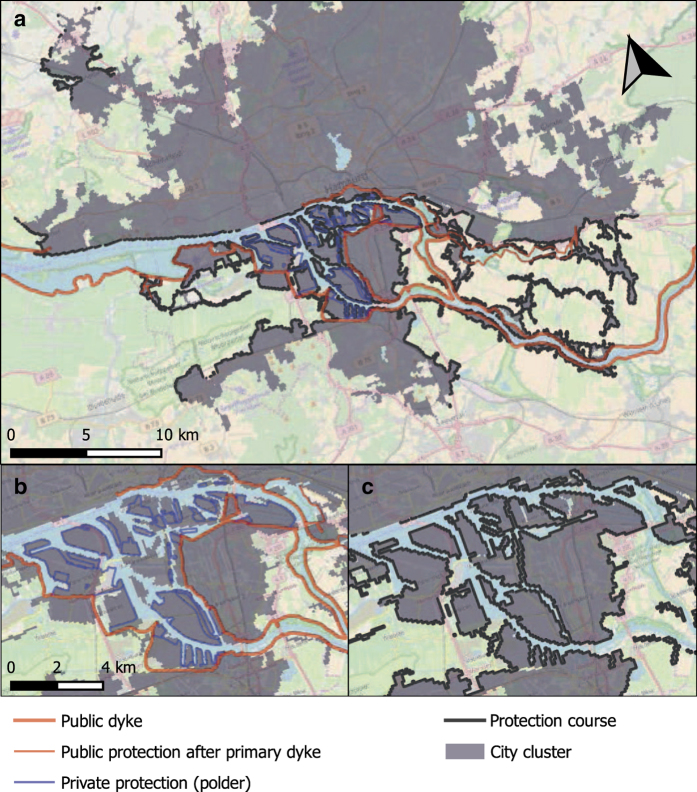
Comparison of the existing protection and the urban protection course (UPC) at 8 m protection height for the city of Hamburg. In Panel (**a**) the existing protection^43^ has been overlain onto the UPC derived from the city cluster. Panels (**b**) and (**c**) show close-ups of the existing protection and the UPC within the city center, respectively.

With a major commercial port on the river Elbe, Hamburg has a longstanding tradition in flood protection. Unfortunately, a quantification of the total cost of its existing protection is not possible due to the diversity of individual protection measures and the many evolutionary steps in reinforcing and readapting to current protection needs. However, official spatial information^43^ enables a comparison between the existing protection measures and the UPC with regard to location and extent. Accordingly, Figure 7a shows the existing protection measures as well as the UPC for an 8 m design level. This approximately compares to the design levels of the existing dikes^43^, which vary from location to location with values mostly fluctuating around 8 m. Perhaps the most striking is the fact that the existing protection closely follows the embankments of the river Elbe, whereas the UPC shows a more ragged behavior and extends into the marshlands and low-lying agricultural lands in the south-west and south-east of the city. This difference is a direct consequence of our modeling choice to protect only those areas that belong to the urban cluster and thus to ensure consistency between the damage cost and protection cost curves.

The close-up of the city center and harbor in Figure 7b reveals a variety of public and private protection measures. Whereas public dikes tend to follow the embankment and protect the entire hinterland, the private polders (defined as low-lying tracts of land that are enclosed by dikes, forming an artificial hydrological entity without natural connections to outside waters) protect selected parts of the harbor. There is a good resemblance of these existing protection measures with the UPC in Figure 7c. The main limitation of the approach is that it is purely cost based, i. e. does not consider the economic benefits from protection. As a consequence, the UPC may enclose urban areas of low economic value that from an economic point of view should remain unprotected. Given that no database of coastal protection location is available for European cities, our results introduce the first approximation to date and provide a basis for discussion of further developments.

In summary, the comparison of protection measures indicates that the UPC is by large consistent with the existing protection measures. The limitations are due to the constricted focus on urbanized areas and the negligence of economic viability.

## Usage Notes

### Vertical reference datum

All cost curves are derived from EU-DEM data and relate to the vertical datum specified by the European Vertical Reverence System 2000 (EVRS2000) with quasi-geoid EGG08 (ref. 44). Note that before applying the supplied damage and protection cost curves it may be necessary to convert the vertical datum of flood-gauge or sea-level data which are typically referenced to the mean sea level (MSL). This aspect has recently been acknowledged in the flood-hazard community and was found to induce large errors and greatly affect flood exposure^45^. A possible bias correction could be based on observations of the mean dynamic ocean topography (MDT), which provides the difference between the mean sea level and the reference geoid^45^.

### Application

The damage and protection cost curves can be the starting point for an economic assessment of coastal flooding impacts and adaptation at the city scale. Together with information on the height of floods at different return periods, they allow for the comparability of the risk from flooding in different cities.

A common approach to obtain the distribution of extreme sea levels is to apply extreme value statistics^46^ to empirical or modeled flood-gauge data^11^. From the distribution, it is possible to infer the return periods of floods at certain height, or vice versa, to obtain the estimated flood height for a given return period.

The damage cost curve can be employed to transform the distribution of (extreme) sea levels to the distribution of damage cost^3^. Specifically,

(3)PD(d)=PX(D−1(d)),

where *P*_*D*_ and *P*_*X*_ are the cumulative distribution functions of the damage *d* and the extreme sea levels *x*, respectively, and *D*^−1^ represents the inverse of the damage cost curve.

Since information on existing coastal defense measures are hardly available consistently for any location^8^ we did not consider existing protection in the cost curves. However, given information on existing protection levels (in terms of protection height or return period), the damage and protection costs curves can be manipulated to reflect the effects of existing protection.

The simplest approach to incorporate existing protection into the damage cost curve would be a truncation of damage cost at the design flood height *z*,

(4)D'(x)={0 ifx<zD(x) ifx≥z,

such that flood events of magnitude smaller than *z* do not cause any damage. Evidently, such a stylized approach falls short of considering the risk of protection failure (e.g. dike breach) and could understate the monetary risk from flood events below the design flood height of the flood defense measure. In order to overcome this limitation, fragility curves^47^ could be employed to model the probability of e.g. dike breaches or wave over-topping.

Similarly, the calculation of the cost of raising or upgrading existing protection *P*' can be directly inferred from the protection cost curves,

(5)P'(ω)={0 ifω<zP(ω)−P(z) ifω≥z. 

In this case, the cost *P* of the already built coastal protection up to flood height *z* is simply subtracted from the total cost at protection height *ω*. The feasibility of this approach hinges on the assumptions made in this work, namely the unit-cost of dike construction and the predetermined protection course at the city cluster boundary. Since the unit-cost approach should be most reliable for the case of dike raise, we recommend to consider only protection cost increments between well-defined protection levels (e. g. upgrading protection levels from a 100-year to a 1000-year return period).

### Adjustment for economic development and urban growth

The damage cost curves provided in this work reflect the socioeconomic conditions of the reference period. This poses no problem if the damage assessment is targeted at the isolated effect of changes in the hazard (e. g. sea level rise or changing storm climates). In contrast, socioeconomic scenarios cannot be considered without making certain changes to the employed damage cost curves. In the following, we argue on the feasibility of adjusting for economic development as well as urban growth and provide some hints how this could be achieved on the basis of the data provided.

Research has shown a strong correlation between economic development (in therms of GDP) and the accumulated asset value^8^. In our work and in similar studies (e.g. ref. 4,8) the relation between GDP and asset value has been used to approximate asset values at different geographical locations. In the same way, the model can be adjusted to reflect future projections of GDP. Such projections are, for example, available from the SSP scenarios developed within the climate-change community^48^. Practically, the damage cost curves should be multiplied by the ratio of future over current GDP per capita.

**Figure 8:**
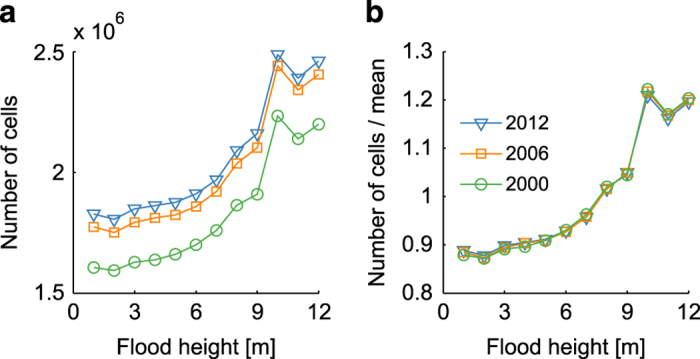
The proportionality of flood-prone urban cells regarding the years 2000, 2006, and 2012. (**a**) Absolute number of urban cells affected at 1 m increments of flood height. (**b**) Number of urban cells divided by the mean over all increments. The estimates for the different years are based one the respective CORINE datasets.

The adjustment for future growth of the urban clusters is less obvious and only possible under certain assumptions. In our view, land-use changes (e.g. from Transport to Industrial) or spatially explicit growth patterns cannot be adjusted for, since these result in specific non-linear transformations of the damage cost curve. However, we suggest that for large-scale assessment urban growth could be considered in a more general statistical manner. In order to motivate this hypothesis, we have computed the number of urban cells in Europe that would be affected at 1 m increments of flood height on the basis of three different time slices of the CORINE data, namely for the years 2000, 2006, and 2012. The results shown in Figure 8a indicate a proportionality between the different years. Accordingly, it should be possible to collapse the curves onto each other by simply dividing by the the mean value over all increments. As seen in Figure 8b, the curves fall nicely onto each other, which confirms the direct proportionality. Comparing the different mean values, we estimate an average growth of 9.7% between 2000 and 2006 and 2.6% between 2006 and 2012. Most importantly, the proportionality, i. e. the constant growth of urban cells at various flood height increments, also implies proportionality of the damage cost for different time slices. As a approximation, one could assume that the spatial growth rate of an urban cluster is the same as for the European or national level. Hence, to approximate a scenario with assumed spatial growth rate *g* the damage cost curves could be multiplied by a factor (1+*g*).

Unfortunately, the protection cost curves provided in this work cannot be adjusted in a similar way. Here, the consideration of urban growth would require a reconstruction of the UPC and recalculation of the protection cost curve, since the UPC is crucially dependent on the geographical pattern of the cluster.

## Additional information

**How to cite this article:** Prahl, B. F. *et al.* Damage and protection cost curves for coastal floods within the 600 largest European cities. *Sci. Data* 5:180034 doi: 10.1038/sdata.2018.34 (2018).

**Publisher’s note:** Springer Nature remains neutral with regard to jurisdictional claims in published maps and institutional affiliations.

## Supplementary Material



Supplementary Information

## Figures and Tables

**Table 1:** Detailed overview of the data sources used in this study.

Type	Details	Reference
Digital Elevation Model of Europe (EU-DEM)	Year 2000, 25 m×25m resolution. The EU-DEM dataset is based on satellite-based SRTM and ASTER GDEM data, which have been fused by a weighted averaging approach. Being a product of interpolation, the vertical resolution of the EU-DEM is variable and given as float numbers in meters.	^13^
EEA coastline for analysis	Year 2013 (updated 2015), coastline for geographical Europe, derived from two sources: EU-Hydro and GSHHG. The criteria for defining the coastline is the line separating water from land.	^14^
CORINE Land Cover (CLC)	Year 2012, 100 m× 100 m resolution. The CLC datasets map homogeneous landscape patterns, i. e. more than 75% of the pattern has the characteristics of a given land-cover class. The class nomenclature is a 3-level hierarchical classification system. In its detailed version it accounts for 44 classes, 8 of which are considered in the derivation of the city clusters according to the UMZ definition^22^.	^16^
Land Use and Coverage Area frame Survey (LUCAS)	Year 2015, harmonized and comparable statistics on land use and land cover for Europe. LUCAS provides statistical data derived from ground surveys carried out in situ, i. e. observations are made and registered in the field all over the EU. Eurostat carried out the latest LUCAS survey between March and October 2015, visiting a total of 273 401 points. LUCAS defines land use in 14 main categories, 6 of which can be mapped to the CLC classes considered in the UMZ definition^22^ for the derivation of city clusters (cf. Table 2)	^17^
Relative damage functions for land-use classes	Year 2004 (updated 2017), land-use-specific damage functions in EU member states. The study comprised a literature review (including questionnaires to the authors), an economical characterization of the countries, and a harmonization procedure which includes the elaboration of maximum damage values for all land-use classes and countries.	^18,19^

**Table 2:** Statistical mapping of CORINE land-cover classes to LUCAS land-use classes, derived from 2015 LUCAS raw data and the 2012 CORINE dataset.

LUCAS class	CORINE class							
	1.1.1 Continuous urban fabric	1.1.2 Discontinuous urban fabric	1.2.1 Industrial or commercial units	1.2.2 Road and rail net- works	1.2.3 Port areas	1.2.4 Airports	1.4.1 Green urban areas	1.4.2 Sport and leisure facilities
U110 Agriculture	0.04	0.22	0.11	0.19	0.01	0.09	0.09	0.15
U220 Industry	0.00	0.01	0.13	0.02	0.10	0.00	0.00	0.00
U310 Transport	0.20	0.14	0.25	0.44	0.53	0.66	0.13	0.09
U340 Commerce	0.05	0.02	0.11	0.02	0.06	0.00	0.02	0.02
U370 Residential	0.49	0.41	0.07	0.01	0.07	0.01	0.14	0.12
U400 Unused	0.05	0.07	0.13	0.20	0.14	0.07	0.11	0.11
Considered share (all of the above)	0.83	0.87	0.80	0.87	0.90	0.84	0.49	0.49
The table shows the observed frequency at which the considered CORINE land-cover classes coincide with those LUCAS land-use classes that we use for damage estimation.Statistically, these frequencies correspond to an average share of a CORINE cell that can attributed to a certain land-use class and are hence used as weights *w*_*i*∧*j*_ in Equation (1). The considered share defines the overall fraction of a land-cover class that can be statistically attributed to the relevant land-use classes. E.g. “Discontinuous urban fabric” corresponds to 22% Agriculture, 1% Industry, 14% Transport, 2% Commerce, 41% Residential, and 7% Unused, accounting for 87% of the total area on average.								

**Table 3:** Statistics on the number of clusters and affected values per country.

Country	# of Clusters	Considered share	Affected at 5 m flood height	
			Value [billion €]	Cluster share
United Kingdom	87^a^	0.81	139.60	0.66
France	68	0.84	102.28	0.58
Netherlands	65	0.81	540.53	0.67
Italy	54	0.84	127.83	0.47
Spain	50	0.82	45.22	0.58
Sweden	39	0.80	46.62	0.57
Germany	34	0.81	139.73	0.46
Denmark	32	0.78	66.90	0.57
Turkey	31	0.81	22.40	0.53
Norway	22	0.83	17.04	0.28
Greece	20	0.83	13.81	0.35
Finland	18	0.83	36.96	0.58
Portugal	14	0.83	5.55	0.50
Belgium	11 (17)^b^	0.84	87.12	0.81
Ireland	10	0.81	14.45	0.56
Poland	8	0.80	16.25	0.50
Cyprus	6	0.81	4.26	0.79
Latvia	5	0.78	16.52	0.70
Albania	4	0.86	2.34	0.51
Bulgaria	4	0.77	1.71	0.62
Croatia	4	0.86	1.97	0.26
Romania	4	0.81	3.44	0.38
Iceland	3	0.77	9.32	0.36
Estonia	2	0.82	1.09	0.36
Lithuania	2	0.82	2.23	0.43
Isle of Man	1	0.82	0.02	0.09
Malta	1	0.84	0.20	0.40
Montenegro	1	0.85	0.19	0.25
For each country, the considered share indicates the fraction of the total cluster area that can be attributed to the considered land-use classes. The affected monetary value is determined by taking into account the considered share for the areas that are affected by a certain flood. We give an example of the affected values at a hypothetical 5 m flood height. The cluster share indicates the share of the affected values in each country that are included in the coastal city clusters of this study.				

^a^Includes the Liverpool/Manchester megacluster. When split, the number of clusters remains unchanged since the Manchester subcluster does not qualify as a coastal cluster.

^b^We obtain 7 coastal subclusters from splitting up the Flemish Diamond.

**Table 4:** Data and methodological differences between this study and the external studies under comparison.

Dataset	This study	Hallegatte *et al.* 2011 (ref. 2)	Hallegatte *et al.* 2013 (ref. 7)
Urban area	Urban cluster from 100 m CORINE land-cover data	Post-code area^a^	Post-code area^a^
Orography	30 m resolution EU-DEM (SRTM based)	90 m SRTM	90 m SRTM, except for proprietary 10 m DEM in the UK
Exposure	Value per m^2^ derived from LUCAS land-use data	Insured value^a^	*Produced capital* based on Landscan 2002 population and GDP per capita
Flooding	Static inundation scheme for hydraulically connected cells	Layers of elevation above MSL	Layers of elevation above MSL
Damage	Relative depth-damage functions for 5 land uses	Vulnerability curves for asset classes residential, commercial, and industrial^a^	Relative depth-damage functions for 6 building and contents classes

^a^Proprietary data owned by Risk Management Solutions (RMS).
