# Supplementary figures and images for: Damage and protection cost curves for coastal floods within the 600 largest European cities
Source: Sci Data. 2018 Mar 20;5:180034. doi: 10.1038/sdata.2018.34 (PMC5859879; doi:10.1038/sdata.2018.34)

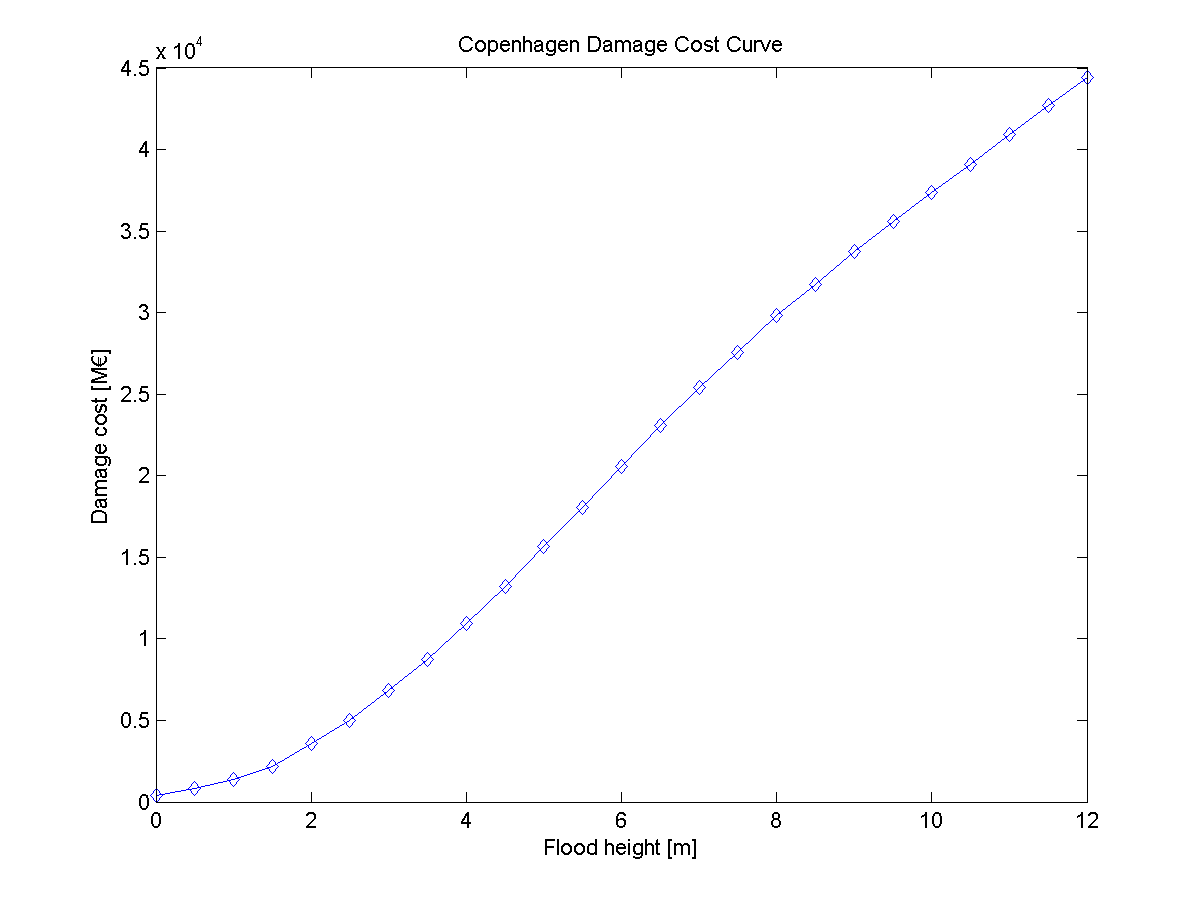

Supplement: Supplementary Information [file sdata201834-s2.zip › damage_cost.png]

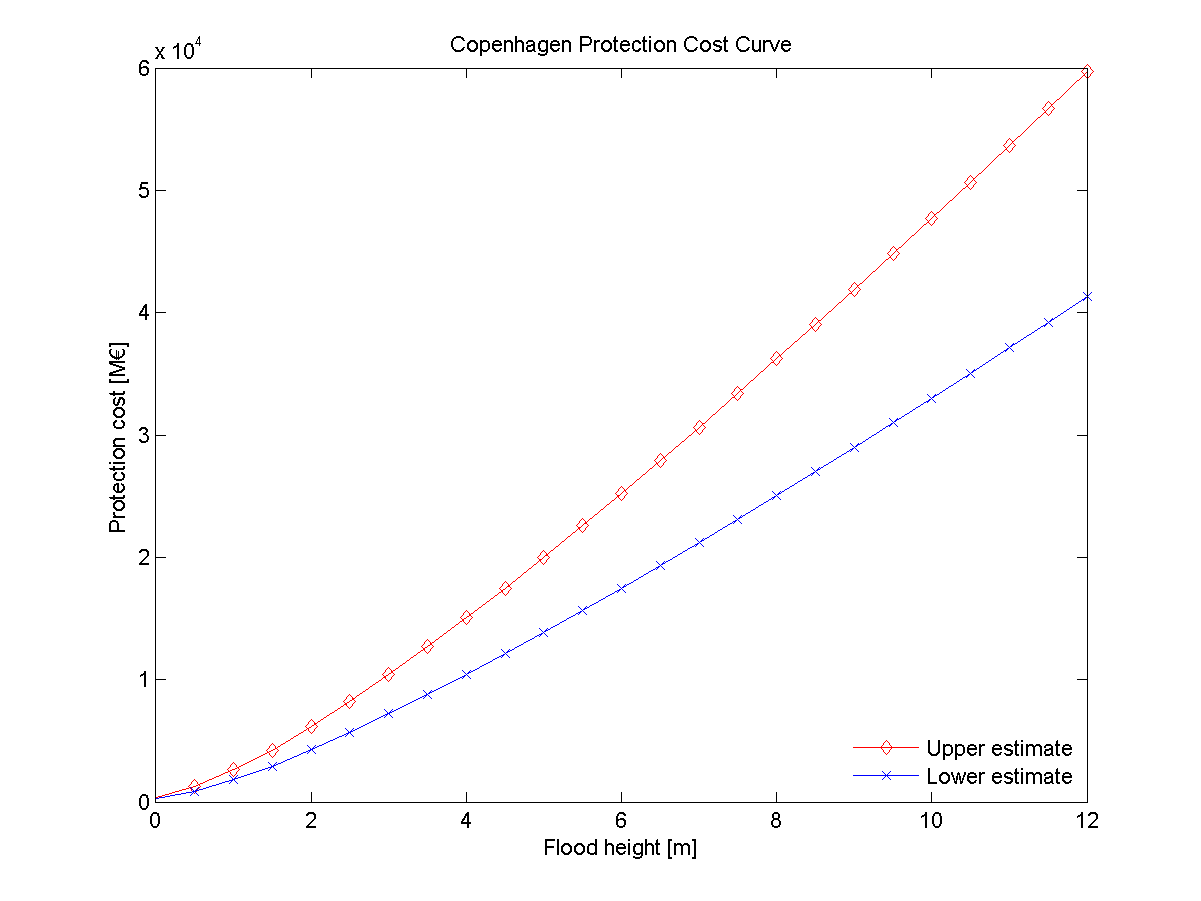

Supplement: Supplementary Information [file sdata201834-s2.zip › protection_cost.png]
